# Use of Intracoronary Pressure Wire Assessment and Association With Outcomes: Insights From the British Cardiovascular Intervention Society National Database

**DOI:** 10.1002/ccd.70835

**Published:** 2026-08-30

**Authors:** Hussein Bashar, Muhammad Rashid, Mamas A. Mamas, Nick Curzen

**Affiliations:** ^1^ Faculty of Medicine University of Southampton Southampton UK; ^2^ Coronary & Structural Heart Research Group University Hospital Southampton NHS Foundation Trust Southampton UK; ^3^ Keele Cardiovascular Research Group, Centre for Prognosis Research Institute for Primary Care and Health Sciences, Keele University Keele UK

**Keywords:** in‐hospital outcomes, intracoronary pressure wire, predictors, procedural complications, trends

## Abstract

**Background:**

Despite recommendations in international guidelines, Intracoronary pressure wire assessment (PWA) utilization remains limited in clinical practice. This study aims to describe (i) trends of PWA during Percutaneous coronary interventional (PCI), (ii) factors that predict its application, and (iii) association with in‐hospital outcomes.

**Methods:**

Adults with PCI for chronic coronary syndrome or Non‐ST‐elevation acute coronary syndrome in the British Cardiovascular Intervention Society (BCIS) database from January 2012 to March 2020 were included. Adjusted odds ratios (aOR) of PWA predictors and its association with in‐hospital major adverse cardiovascular and cerebrovascular events (MACCE; a composite of total mortality, stroke/transient ischaemic attack, and new/reinfarction) were assessed using logistic regression models.

**Results:**

303,453 records identified of which 10.74% had PWA. There was a modest rise in the use of PWA from 9.1% in 2012 to peak just over 13% in 2018. Intracoronary imaging (aOR: 1.23, 95% CI: 1.18–1.27, *p* < 0.001), and previous PCI (aOR: 1.30, 95% CI: 1.26–1.34, *p* < 0.001) were positive predictors of PWA. MACCE was lower with PWA (aOR: 0.73, 95% CI: 0.62–0.87, *p* < 0.001) in the overall population. PWA was associated with lower procedural complications (aOR: 0.90, 95% CI: 0.84–0.96, *P*: 0.001).

**Conclusion:**

PWA remains underutilized in UK real world practice. Among patients undergoing PCI, PWA was associated with lower in‐hospital procedural complications and MACCE. It's association with in‐hospital MACCE should be viewed as a hypothesis‐generating for future studies. Using pressure wire as a performance and a quality metric for PCI might help improve adoption in the future.

## 1 Introduction

There is a large body of evidence to support the use of intracoronary pressure wire assessment (PWA) to assess the functional significance of coronary lesions and that this approach is associated with improved outcomes when used to direct percutaneous coronary intervention (PCI) compared with angiographic guidance alone [1, 2]. This level of evidence is reflected in international clinical practice guidelines [3, 4]. Despite this, adoption of this technology in real world practice remains inconsistent, and lower than expected overall. This could be related to a variety of factors including concerns about perceived extra procedural time, risk and costs as well as concerns that pressure wire use may reduce rates of PCI. There is also awareness of the consistently neutral results of randomised control trials for hard clinical endpoints that have used routine pressure wire of the coronary circulation for patient diagnosis and management, including RIPCORD2, FUTURE, FAME3 and FLOWER‐MI [5, 6, 7, 8].

The use of PWA as a percentage of overall PCI rates is highly variable both between operators and centres, but there is limited data around patterns of pressure wire adoption and the predictors of pressure wire use particularly from a national perspective, and whether there is an association between use of PWA in PCI cases and in hospital outcomes, particularly given that previous data from this database indicated an association between intracoronary imaging and outcome [9]. The aims of this study are to describe (i) trends in the use of PWA during PCI in the United Kingdom, (ii) factors that predict its application, and (iii) any association between use of PWA with in‐hospital outcomes.

## 2 Methods

### 2.1 Data Source, Study Sample and Design

Percutaneous coronary interventional procedure records from January 2012 to March 2020 (latest available at the time of the analysis) were uploaded from the British Cardiovascular Intervention Society (BCIS) database. This database is a registry of all PCI procedures performed in England and Wales that includes clinical, procedural and in‐hospital outcome data [10]. Adults (aged ≥ 18 years), in whom PCI was attempted for chronic coronary syndrome (CCS) or Non‐ST‐elevation acute coronary syndrome (NSTE‐ACS) were included in this analysis. Only cases in which PCI was performed or attempted were included in this analysis, so that diagnostic‐only pressure wire cases were excluded. Missing data as well as legacy physiology and imaging devices were excluded (Figure S2). Procedures where stents were not used (i.e. diagnostic only pressure wire cases) were also excluded.

The study sample was stratified into two groups based on the presence/absence of physiological assessment, including both fractional flow reserve (FFR) or Instantaneous wave‐free ratio (iFR).

### 2.2 Outcomes

The primary clinical outcome of this analysis was major adverse cardiovascular and cerebrovascular events (MACCE; defined as a composite of total mortality, stroke or transient ischaemic attack (TIA), and new/reinfarction). Individual components of the primary outcome as well as major bleed, re‐intervention, procedural and arterial access complications were included as secondary outcomes. Major bleed was defined as type three or higher on the Bleeding Academic Research Consortium (BARC) classification [11]. Procedural complications included any of: side branch loss, coronary dissection, aortic dissection, coronary perforation, heart block requiring pacing, direct current cardioversion, no flow/slow flow phenomenon, mechanical ventilation, shock, or other undefined complications. Arterial access complications encompassed false aneurysm, dissection, occlusion, needing surgical intervention, bleeding delaying‐discharge, hemorrhage with no hematoma, and retroperitoneal bleeding.

### 2.3 Statistical Analysis

All statistical analysis were performed using the IBM SPSS statistics software version 29.0.1.0. Age and number of stents variables were transformed into categorical values to facilitate analysis. All presented variables are categorical, given as percentage, and compared using the Pearson's chi square test.

Pre‐procedure renal failure is defined as either creatinine of greater than 200 micromole/Liter or dialysis for acute/chronic kidney disease, by the BCIS registry. Glycoprotein IIb/IIIa inhibitors use included any of Abciximab, Eptifibatide, and Tirofiban. Intracoronary imaging described the use of intravascular ultrasound (IVUS) or optical coherence tomography (OCT). The utilization of directional atherectomy, rotational atherectomy, cutting balloon, laser angioplasty, or shockwave balloon was regarded as calcium modification. Angiographic disease severity was classified as mild (< 50% stenosis), moderate (50%–74% stenosis), severe (75%–99% stenosis, ≥ 50%–99% stenosis for left main stem, LMS), and total occlusion.

The predictors for physiological assessment utilization as well as its relationship with in‐hospital outcomes were presented as adjusted odds ratios (aOR) with their corresponding 95% confidence interval (CI). These were determined using multivariable binomial logistic regression models that were adjusted for: age group, sex, previous myocardial infarction (MI), previous coronary artery bypass graft surgery (CABG), previous PCI, diabetes mellitus, smoking history, family history of coronary artery disease, dyslipidaemia, hypertension, peripheral vascular disease, previous stroke, cardiac transplant, valvular heart disease, previous non‐coronary cardiac surgery, renal function, aspirin, clopidogrel, warfarin, prasugrel, ticagrelor, clinical presentation, glycoprotein IIb/IIIa inhibitors use, per vessel angiographic disease severity, intra‐coronary imaging, calcium modification, attempted vessel, and arterial access. These models did not account for left ventricular ejection fraction, stent thrombosis data, and race/ethnicity due to the large proportion of unknown values in these variables. Sensitivity analysis of the forementioned outcomes was conducted among a sub‐selected population with CCS.

## 3 Results

A total of 303,453 attempted PCIs with at least one stent deployed for CCS/NSTE‐ACS were included in this analysis. Data with PWA represented 10.74% of the cases (32,597). There were 136,649 PCI records for CCS in the sensitivity analysis, of whom 21,190 did have PWA (15.51%).

### 3.1 Temporal Trends

There was a modest rise in the use of PWA from 9.1% in 2012 which peaked just over 13% in 2018 before declining to less than 12% in 2020 (Figure 1). During the same time period, Intracoronary imaging underwent rapid growth from 7.3% in 2012 to over 20% by 2020. In the CCS population, PWA use rose from just over 13% in 2012 to the highest point of 19.3% in 2018 then trended down to 17.4% in 2020 (Figure S3). In both samples, PWA use rate declined after 2018, with the incidence of angiographically moderate (50%–74% stenosis) coronary artery disease (CAD) after 2018 declining form over 30% to more than 28% in both samples.

**Figure 1:**
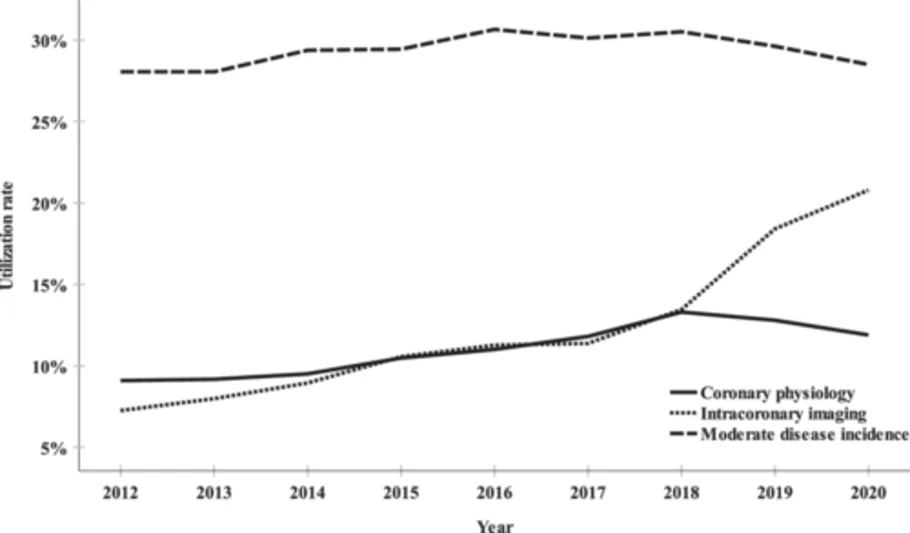
Utilization trends of coronary physiology and intracoronary imaging during PCI.

Examining PWA utilization rate by the presence of moderate coronary stenosis shows a decline in its application for this indication toward the end of the study period (from a peak of 7.3% in 2018% to 6.2% in 2020) (Figure 2) which can also be observed in the stable angina population (highest of 10.3 in 2018 to 8.7 in 2020) (Figure S4) but with a modest rise in its use in the absence of angiographic moderate CAD in 2019.

**Figure 2:**
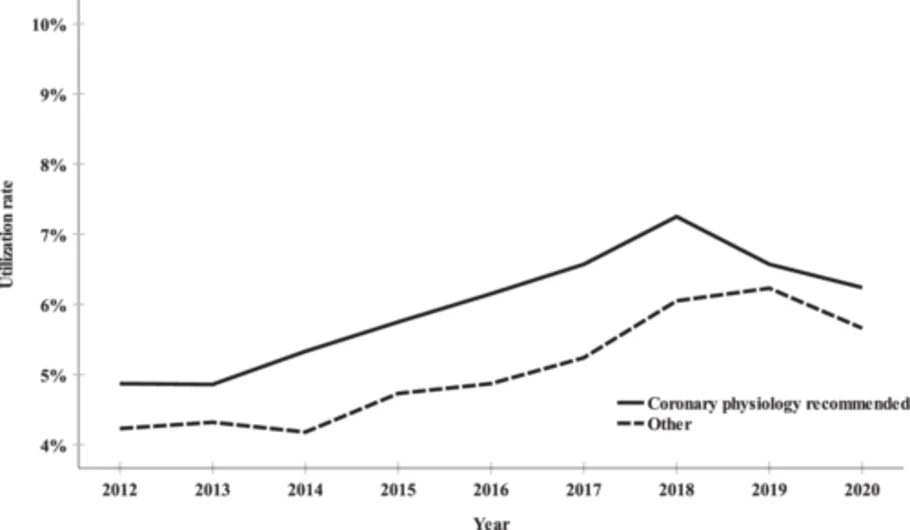
Utilization trends of coronary physiology stratified by the presence of guideline recommendation for coronary physiological assessment (I.e., the presence of moderate disease).

### 3.2 Baseline and Procedural Characteristics

In comparison with those who didn't have physiological assessment, patients in the PWA arm of this analysis were more likely to have had previous MI (32.56% vs. 30.52, *p* < 0.001), previous PCI (38.08% vs. 28.84%, *p* < 0.001), CCS presentation (65.01% vs. 42.63%, *p* < 0.001), Intracoronary imaging use (14.50% vs. 10.86%, *p* < 0.001), and radial access (86.28% vs. 79.21%, *p* < 0.001) (Table 1).

**Table 1:** Baseline and procedural characteristics.

Characteristics, %	No physiological assessment (*N*= 270,856; 89.26%)	Physiological assessment (*N*= 32,597; 10.74%)	*p*‐value
Age group (years)			< 0.001
< 60	31.01	33.52	
60–69	29.28	32.64	
70–79	26.74	26.01	
≥ 80	12.98	7.83	
Male	74.07	76.75	< 0.001
Race/Ethnicity			
Caucasian	60.77	56.79	
Black	0.63	0.49	
Asian	5.59	4.95	
Oriental	0.13	0.13	
Other	5.76	5.91	
Unknown	27.14	31.72	
Previous MI	30.52	32.56	< 0.001
Previous CABG	9.68	3.97	< 0.001
Previous PCI	28.84	38.08	< 0.001
DM	23.60	23.06	0.028
Smoking history	59.34	59.21	0.638
Family history of CAD			< 0.001
None	51.05	47.64	
Known	42.28	45.87	
Unknown	6.67	6.49	
Dyslipidaemia	56.22	60.85	< 0.001
Hypertension	59.03	61.33	< 0.001
PVD	4.85	3.93	< 0.001
Previous stroke	4.38	4.15	0.063
Cardiac transplant	0.08	0.06	0.418
Valvular heart disease	2.18	1.98	0.020
Previous non‐coronary cardiac surgery	0.56	0.47	0.029
Renal function			< 0.001
No renal disease	97.01	97.95	
Functional transplant	0.29	0.27	
Renal failure	2.69	1.78	
LV ejection fraction			
Good (> 50%)	44.60	52.74	
Moderate (30%–50%)	12.84	9.80	
Poor (< 30%)	3.27	2.14	
Unknown	39.29	35.31	
Pre‐Procedure medications			
Aspirin	87.44	88.60	< 0.001
Clopidogrel	64.32	66.53	< 0.001
Ticagrelor	23.68	22.40	< 0.001
Prasugrel	1.63	1.66	0.723
Warfarin	1.27	1.26	0.942
Procedure characteristics			
Stable angina presentation	42.63	65.01	< 0.001
Intracoronary imaging	10.86	14.50	< 0.001
Calcium modification	7.72	6.68	< 0.001
GPIIb/IIIa inhibitors	15.72	11.16	< 0.001
Stent thrombosis			
Present	0.63	0.32	
Unknown	35.55	31.10	
Arterial access			
Femoral	22.55	14.83	< 0.001
Radial	79.21	86.28	< 0.001
Other	0.37	0.35	0.593
Attempted vessels			
Left main stem	6.19	3.97	< 0.001
Proximal LAD	31.28	37.54	< 0.001
Other LAD	29.17	43.84	< 0.001
LCx	27.76	29.11	< 0.001
RCA	33.69	30.08	< 0.001
Graft	2.91	0.60	< 0.001

Abbreviations: CABG, coronary artery bypass graft; CAD, coronary artery disease; DM, diabetes mellitus; LV, Left Ventricle; MI, myocardial infarction; PCI, percutaneous coronary intervention; PVD, peripheral vascular disease.

Patients who didn't undergo PWA included a higher proportion of older adults (aged 80 years or older, 12.98% vs. 7.83%, *p* < 0.001), previous CABG (9.68% vs. 3.97%, *p* < 0.001), renal failure (2.69% vs. 1.78%, *p* < 0.001), with higher burden of angiographically severe CAD (Table 1 and Table S5), and attempted PCI to the LMS (6.19% vs. 3.97%, *p* < 0.001).

In sensitivity analysis of patient with CCS, the baseline and procedural characteristics distribution between the two arms of the study were generally similar to that of the whole research population with exception of a higher percentage of previous MI (35.02% vs. 33.97%, P 0.003), and a lower proportion pretreated with Ticagrelor (13.70% vs. 15.24%, *p* < 0.001), in those who did not undergo PWA in comparison to those who had coronary physiology analysed (Table S6).

### 3.3 Predictors of Coronary Physiological Assessment

All moderate angiographic stenoses were positive predictor of PWA with proximal LAD moderate disease being the strongest (aOR: 2.38, 95% CI: 2.28–2.49, *p* < 0.001). Mild proximal LAD (aOR: 1.28, 95% CI: 1.24–1.33, *p* < 0.001) as well as mild RCA (aOR: 1.08, 95% CI: 1.04–1.12) stenosis were also positive predictors of PWA. Other robust positive predictors of PWA use include previous PCI (aOR: 1.30, 95% CI: 1.26–1.34, *p* < 0.001), Intracoronary imaging (aOR: 1.23, 95% CI: 1.18–1.27, *p* < 0.001) and attempted LAD PCI (Table 2).

**Table 2:** Predictors of coronary physiological assessment.

Predictor	aOR [95% CI]	*p*‐value
Age group (years)		
< 60	Reference	
60–69	0.97 [0.94–1.00]	0.035
70–79	0.87 [0.85–0.90]	< 0.001
≥ 80	0.60 [0.57–0.63]	< 0.001
Male	1.08 [1.05–1.11]	< 0.001
Previous MI	1.01 [0.97–1.04]	0.702
Previous CABG	0.89 [0.83–0.95]	< 0.001
Previous PCI	1.30 [1.26–1.34]	< 0.001
DM	1.00 [0.97–1.03]	0.922
Smoking history	1.02 [1.00–1.05]	0.080
Family history of CAD		
None	Reference	
Known	1.06 [1.03–1.08]	< 0.001
Unknown	1.05 [1.00–1.11]	0.050
Dyslipidaemia	1.06 [1.03–1.09]	< 0.001
Hypertension	1.08 [1.05–1.11]	< 0.001
PVD	0.95 [0.89–1.01]	0.125
Previous stroke	1.06 [1.00–1.13]	0.049
Cardiac transplant	0.86 [0.54–1.37]	0.526
Valvular heart disease	1.09 [1.00–1.19]	0.058
Previous non‐coronary cardiac surgery	0.92 [0.77–1.10]	0.379
Renal function		
No renal disease	Reference	
Functional transplant	0.98 [0.78–1.24]	0.868
Renal failure	0.82 [0.75–0.90]	< 0.001
Pre‐Procedure medications		
Aspirin	1.10 [1.05–1.15]	< 0.001
Clopidogrel	0.99 [0.95–1.03]	0.471
Ticagrelor	1.06 [1.02–1.10]	0.007
Prasugrel	0.82 [0.74–0.90]	< 0.001
Warfarin	0.98 [0.88–1.10]	0.743
Procedure characteristics		
Clinical Presentation		
CCS	Reference	
NSTE‐ACS	0.42 [0.41–0.43]	< 0.001
Intracoronary imaging	1.23 [1.18–1.27]	< 0.001
Calcium modification	0.78 [0.74–0.82]	< 0.001
GPIIb/IIIa inhibitors	0.79 [0.76–0.82]	< 0.001
Arterial access		
Femoral	0.79 [0.74–0.84]	< 0.001
Radial	1.24 [1.16–1.32]	< 0.001
Other	1.19 [0.96–1.47]	0.104
Angiographic severity		
Left main stem		
Normal	Reference	
Mild disease	1.02 [0.99–1.06]	0.216
Severe disease	0.78 [0.71–0.86]	< 0.001
Totally occluded	0.98 [0.62–1.56]	0.929
Proximal LAD		
Normal	Reference	
Mild disease	1.28 [1.24–1.33]	< 0.001
Moderate disease	2.38 [2.28–2.49]	< 0.001
Severe disease	0.76 [0.72–0.80]	< 0.001
Totally occluded	0.40 [0.36–0.45]	< 0.001
Other LAD		
Normal	Reference	
Mild disease	1.06 [1.02–1.10]	0.003
Moderate disease	2.08 [2.00–2.17]	< 0.001
Severe disease	0.80 [0.77–0.84]	< 0.001
Totally occluded	0.39 [0.36–0.43]	< 0.001
LCx		
Normal	Reference	
Mild disease	1.01 [0.98–1.05]	0.437
Moderate disease	1.53 [1.46–1.60]	< 0.001
Severe disease	0.81 [0.77–0.86]	< 0.001
Totally occluded	0.72 [0.67–0.77]	< 0.001
RCA		
Normal	Reference	
Mild disease	1.08 [1.04–1.12]	< 0.001
Moderate disease	1.87 [1.79–1.96]	< 0.001
Severe disease	0.83 [0.79–0.88]	< 0.001
Totally occluded	0.52 [0.49–0.55]	< 0.001
Attempted vessels		
Left main stem	0.80 [0.73–0.87]	< 0.001
Proximal LAD	1.55 [1.49–1.62]	< 0.001
Other LAD	2.06 [1.98–2.14]	< 0.001
LCx	1.52 [1.45–1.59]	< 0.001
RCA	1.33 [1.27–1.40]	< 0.001
Graft	0.81 [0.69–0.95]	0.012

Abbreviations: aOR, Adjusted odds ratio; CABG, coronary artery bypass graft; CAD, coronary artery disease; CCS, chronic coronary syndrome; DM, diabetes mellitus; LAD, Left Anterior Descending Artery; LCx, Left Circumflex Artery; LV, Left Ventricle; MI, myocardial infarction; NSTE‐ACS, Non‐ST‐segment Elevation Acute Coronary Syndrome; PCI, percutaneous coronary intervention; PVD, peripheral vascular disease; RCA, Right Coronary Artery.

On the other hand, NSTE‐ACS was the strongest preprocedural negative predictor of PWA (aOR: 0.42, 95% CI: 0.41–0.43, *p* < 0.001). Further, the oldest age groups (70–79, aOR: 0.87, 95% CI: 0.85–0.90, *p* < 0.001; ≥80 years, aOR: 0.60, 95% CI: 0.57–0.63, *p* < 0.001), renal failure (aOR: 0.82, 95% CI: 0.75–0.90, *p *< 0.001), and femoral arterial access (aOR: 0.79, 95% CI: 0.74–0.84, *p* < 0.001) were all associated with decreased odds of PWA application. Angiographically, the presence of severe or totally occluded coronary artery as well as attempted PCI to the LMS were also negative predictors of PWA use as detailed in Table 2.

The predictors of PWA utilization in the sensitivity analysis among patients with chronic coronary syndrome were comparable to that of the general analysis population (Table S7).

### 3.4 Outcomes‐Unadjusted

Compared to procedures with PWA, unadjusted data demonstrate a significantly higher rate of MACCE (1.01% vs. 0.44%, *p* < 0.001) among cases that did not undergo PWA. Similarly, mortality (0.69% vs. 0.13%, *p* < 0.001), procedural complications (3.99% vs. 3.51%, *p* < 0.001) were significantly higher in this arm of the analysis as well as arterial access complications (0.70% vs. 0.38%, *p* < 0.001) and the use of higher number of stents (Table 3).

**Table 3:** Unadjusted in‐hospital outcomes.

Outcomes, %	No physiological assessment (*N* = 270,856; 89.26%)	Physiological assessment (*N* = 32,597; 10.74%)	*p*‐value
MACCE	1.01	0.44	< 0.001
Mortality	0.69	0.13	< 0.001
Stroke/TIA	0.08	0.08	0.937
Re or new infarction	0.27	0.24	0.375
Major bleed	0.24	0.15	0.002
Re‐intervention	0.18	0.16	0.351
Procedural complications	3.99	3.51	< 0.001
Arterial access complications	0.70	0.38	< 0.001
Two or more stents	44.80	44.65	0.594
Three or more stents	16.84	15.80	< 0.001
Four or more stents	5.82	5.09	< 0.001

*Note:* In unadjusted sensitivity analysis of the CCS population, the rate of the primary outcome was not significantly different in both arms of the study (MACCE, 0.51% vs. 0.41%, *p* < 0.054), with higher mortality in the non‐PWA group (0.18% vs. 0.07%, *p* < 0.001). By contrast, procedural complications (3.84% vs. 3.46%, *p* < 0.001), and arterial access complications (0.82% vs. 0.36%, *p* < 0.001) and multiple stents use were higher in the non‐PWA arm (Table S8).

Abbreviations: MACCE, major adverse cardiovascular and cerebrovascular events; TIA, Transient Ischemic Attack.

### 3.5 Outcomes‐Adjusted

After adjustment for patient and procedural characteristics, the PWA group had significantly lower risk of MACCE (aOR: 0.73, 95% CI: 0.62–0.87, *p* < 0.001) and mortality (aOR: 0.41, 95% CI: 0.30–0.56, *p* < 0.001) (Figure 3 and Table 4). By contrast, the risk of procedural complications, and arterial access complications as well as multiple stents deployment were all significantly lower among patients who had PWA (Figure 4).

**Figure 3:**
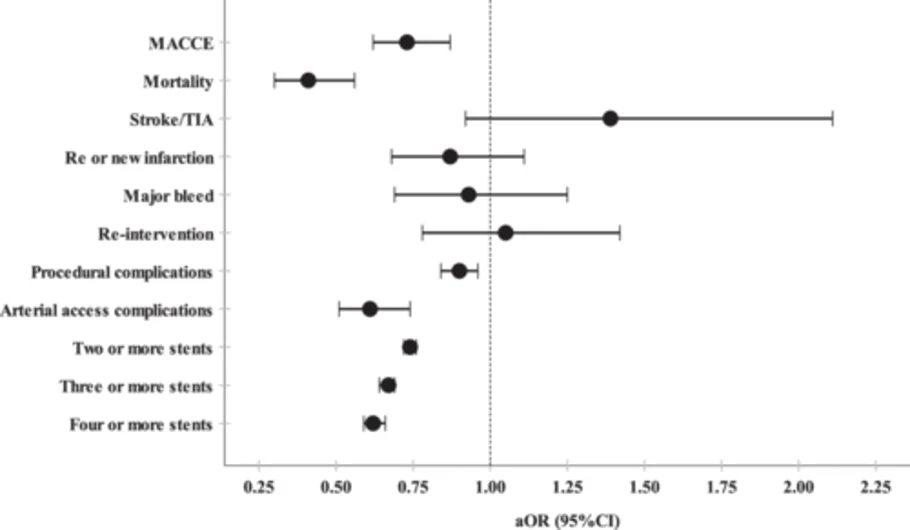
Adjusted odds ratio of in hospital outcomes.

**Table 4:** Adjusted outcomes.

Hospital outcomes	Physiological assessment aOR [95% CI]	*p*‐value
MACCE	0.73 [0.62–0.87]	< 0.001
Mortality	0.41 [0.30–0.56]	< 0.001
Stroke/TIA	1.39 [0.92–2.11]	0.122
Re or new infarction	0.87 [0.68–1.11]	0.259
Major bleed	0.93 [0.69–1.25]	0.621
Re‐intervention	1.05 [0.78–1.42]	0.734
Procedural complications	0.90 [0.84–0.96]	0.001
Arterial access complications	0.61 [0.51–0.74]	< 0.001
Two or more stents	0.74 [0.72–0.76]	< 0.001
Three or more stents	0.67 [0.64–0.69]	< 0.001
Four or more stents	0.62 [0.59–0.66]	< 0.001

*Note:* *Reference group: No physiological assessment. Sensitivity analysis for adjusted outcomes in patients with CCS, PWA was no longer associated with MACCE (aOR: 0.90, 95% CI: 0.71–1.14, *P*: 0.371), and mortality (aOR: 0.59, 95% CI: 0.35–1.01, *p* < 0.056) in this population. Procedural complications (aOR: 0.90, 95% CI: 0.83–0.98, *P*: 0.013), arterial access complications (aOR: 0.54, 95% CI: 0.43–0.69, *p* < 0.001), as well as multiple stents placement were lower in the PWA group (Figure 4 and Table S9).

Abbreviations: aOR, Adjusted odds ratio; CI, confidence interval, MACCE, major adverse cardiovascular and cerebrovascular events; TIA, Transient Ischemic Attack.

**Figure 4:**
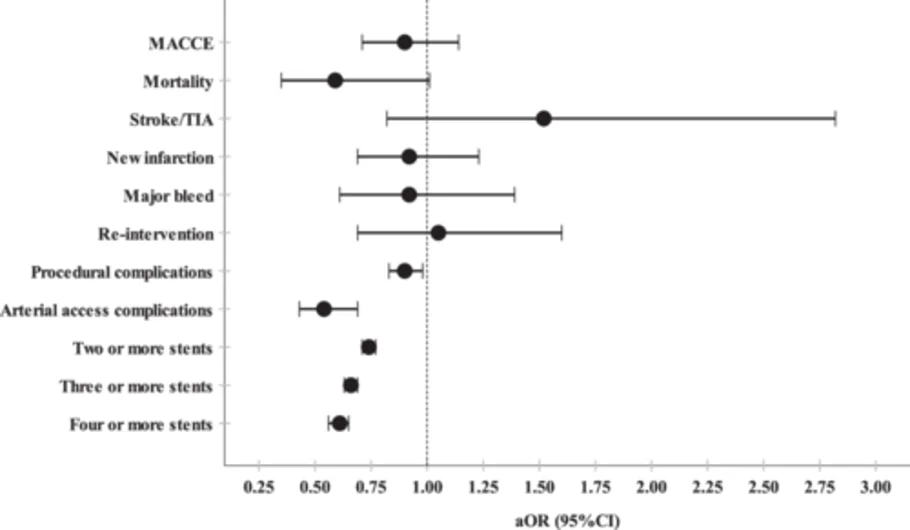
Adjusted odds ratio of in hospital outcomes in patients with stable angina.

## 4 Discussion

This registry‐based analysis of nationwide, real‐world PCI practice in the UK presents the following findings. First, use of PWA has increased by only a modest extent over the study period and remains lower than would be expected according to recommended international clinical guidelines. Second, while moderate stenosis is expected positive predictor of PWA, mild CAD, intracoronary imaging, and previous PCI were also positive predictors of PWA use. Third, PWA was associated with 27% lower adjusted in‐hospital MACCE, that was mainly driven by lower mortality, though this association was not observed in the chronic coronary syndrome population, and thus potentially susceptible to confounding by indication. Fourth, PWA use was associated with 10% lower procedural complications, 39% lower arterial access complications, and lowered the odds of having multiple stents by 26%.

The current data demonstrate that the use of pressure wire in association with PCI has increased over the study period, but the overall numbers remain relatively low. This is consistent with the latest BCIS published audit return of all PWA utilization (including diagnostic only cases) demonstrating a continued decline in PWA from around 19% use in 2019 to less than 15% of all PCI activity in 2023 [12]. In comparison, analysis from a high‐volume Korean PCI centre registry demonstrated a PWA rate of more than 50% [13]. Likewise, PWA utilization has been around 30% in Belgium, Denmark, and Sweden [14, 15, 16, 17]. In the United states National Cardiovascular Data Registry's CathPCI Registry (NCDR CathPCI), the utilization rate of PWA for intermediate stenosis reached 30.8% in 2014 [18] and data from Veterans Affairs Clinical Assessment, Reporting, and Tracking (CART) Program, demonstrating 18.5% PWA use rate in CCS with moderate stenosis in 2017 [19]. In our current study, even when moderate coronary stenosis has been documented, PWA use was low, confirming poor penetrance of this technology in real world practice [14, 20, 21]. Unfortunately, the BCIS database doesn't capture the rational for this. However, this may represent a wider adoption of non‐invasive assessment methods such as CT_FFR_, stress CMR, and stress echocardiography. It's also worth highlighting that the “neutral” pressure wire studies such as FLOWER‐MI, RIPCORD2, and FAME3 were all published after 2020 [5, 8, 22], and thus cannot justify the downtrend in PWA observed in this analysis. However, concerns from the prematurely halted FUTURE trial might have played a role [6]. FUTURE was examining FFR versus angiography guide management in patients with multivessel disease and was prematurely discontinued due to safety concerns indicating higher mortality with FFR. This information was available as early as 2016 prior to the publication of the study later in 2021. However, after full analysis of the trial data, the difference in deaths was nonsignificant. Similarly, the COVID19 pandemic was only confirmed in the UK in late January 2020 with public health measures introduction 2 months later [23], while this paper is limited to the available dataset up to March 2020. Therefore, the utilization trend observed in this analysis is not fully explained. However, this could indicate a practice shift toward using intracoronary imaging, measuring minimal luminal area to guide revascularization decision.

Pressure wires and intracoronary imaging devices address different aspect of coronary artery disease assessment. These technologies can be considered complementary to each other. In an older previous analysis of rotational atherectomy PCI, pressure wire use was a positive predictor of ICI (aOR: 1.51, 95% CI: 1.13–2.03) [24]. This relationship seems to be bidirectional as the current paper demonstrates that ICI is a positive predictor of PWA use (aOR: 1.23, 95% CI: 1.18–1.27, *p* < 0.001). In addition, there have been attempts to correlate the minimal luminal area from ICI and coronary FFR to avoid the use of dedicated PWA, however the data demonstrated variation in the cut off based on the population as well as weak to moderate association with the FFR value, highly dependent on the vessel size and lesion location [25, 26, 27, 28]. Certainly, the field of computational fluid dynamics using ICI to estimate coronary lesion physiological significance is highly promising with much better correlation with invasive data [29, 30], but it remains far from ready for mainstream use to justify the lower than expected uptake in PWA.

A pattern of coronary physiological utilization is also demonstrated that favours its application in non‐severe coronary artery disease, including mild disease. This form of utilization could result in an overall net effect of disease severity upgrade, rather than downgrading significance with pressure wire use. In addition, in‐stent restenosis angiographically often has poor correlation with functional assessment [31], Hence, previous PCI was found as positive predictor of PWA use likely to evaluate for ISR especially when trying to avoid multiple layers of stents. These positive predictors can be indicative of high‐quality operators who are keen on objective assessment methods during revascularization decision‐making.

In addition, recently published observational study from US population demonstrated lower rates of the composite of (myocardial infarction, repeat revascularization, and all‐cause mortality) at 2 years with the combined use of PWA and ICI (adjusted HR: 0.87; 95% CI: 0.85–0.89; *p* < 0.0001) compared with angiography alone [32], which might represent an indirect indicator of operator quality.

The biggest driver for our observation that the adjusted MACCE rate was lower in the PWA group was mortality. These data are consistent with the long‐term mortality data from the SCAAR (Swedish Coronary Angiography and Angioplasty Registry) in both stable angina and ACS population [15, 33], Interestingly, however, the SCAAR investigators did not find any significant difference in mortality during in‐hospital stay. The mechanism for lower MACCE & mortality rate in the pressure wire group from these data could not be fully explained. It is expected from both randomised trial and observational studies in the past that pressure wire use would be associated with fewer stent procedures with less stents (as demonstrated in the current analysis) and a lower rate of major adverse cardiac events [13, 34]. However, there are few indications that pressure wire use is associated with a reduction in mortality, as reported here. However, the primary outcome incidences were overall low in both arms, and the observed difference was not significant in the sensitivity analysis once NSTE‐ACS cases were excluded. NSTE‐ACS population is highly heterogeneous in term of in‐hospital outcomes contingent on other confounders that could not be adjusted for in this study such as troponin levels, ST segment changes, left ventricular function, thrombus burden, time to intervention, surgical turndown, and timing of the admission.

This finding must be contextualized with the contemporary evidence from randomized trials. The FLOWER‐MI study didn't show a significant benefit of FFR guided revascularization of non‐culprit lesions in STEMI patients after successful PCI of the infarct‐related artery, at one as well as 3 years of follow up versus angiography guided non‐culprit revascularization [8, 35]. However, FLOWER‐MI was relatively small (1170 cases) for the size of the effect detected in this BCIS analysis. In addition, FLOWER‐MI included only STEMI patients, in which FFR could be influenced by associated microvascular dysfunction, who experienced higher adverse cardiovascular events when PCI was deferred based on FFR [36]. Similarly, the prematurely stopped FUTURE trail recruited all comers with multivessel disease and didn't demonstrate difference in ischemic cardiovascular events or mortality after 1 year of follow up [6]. FUTURE did include STEMI patients where FFR might be inaccurate in the non‐culprit vessel [37, 38], and had CABG as treatment option which often excel in the long term which this BCIS analysis doesn't examine. Likewise, RIPCORD2 did examine routine FFR of all coronary arteries, of a size suitable for stent or graft, versus angiography only‐guided management of patients with CCS or NSTE‐ACS and showed no differences in cost or quality of life, nor in clinical outcomes [5]. On the other hand, the SLIM and the FIRE trials both showed superior clinical outcomes with PWA‐guided complete revascularization in NSTE‐ACS patients versus culprit only PCI (which allowed non‐culprit PCI only if deemed necessary based on angiography). Of course, however, this might be related to the overall benefit of complete revascularization rather than physiological guidance in particular [39, 40].

The lower procedural complication rate in the PWA group in the current study is consistent with previous observation data [13, 34]. Importantly, the incidence of periprocedural myocardial infarction [41], has been reported to be lower in cases with physiology guided PCI in real world data [13].

Given the finding of this analysis which adds to the large body of evidence behind pressure wires utility, stakeholders should consider the use of pressure wire as a performance and quality metric for PCI, recommending expanding the BCIS database to include data on PWA type, ischaemia, vessel, vessel size, and post PCI residual ischaemia, to improve the penetrance of coronary physiology.

This study has several important limitations largely due to its observational nature. First, several comorbidities, therapeutics, stent thrombosis data and echocardiographic findings remain unaccounted for which can confound the results. Specifically, it is not possible to know why the operator decided to use pressure wire in a particular case and whether there was an unidentified selection bias/confounding by indication. Second, the BCIS database doesn't capture which vessel and lesion was assessed by pressure wire and particularly if that assessment was implemented in their decision‐making regarding revascularization. Nor can it tell if there was residual ischemia after PCI. It also doesn't capture whether the vessel that underwent PCI had the pressure wire assessment or whether it was a different lesion that was managed medically. Third, this analysis only pursues short term in‐hospital outcomes and cannot be extrapolated to forecast long term differences. Finally, the design of this study does not predict any causal relationship, but only association between pressure wire use and in‐hospital outcomes.

In conclusion, among patients undergoing PCI, PWA remains underutilized in UK real world practice. PWA is associated with lower in‐hospital procedural complications and lower multiple stents use. Its association, in the context of PCI, with in‐hospital MACCE and mortality should be viewed as a hypothesis‐generating, and should be the stimulus for prospective future studies. Given these data, it is conceivable that the use of pressure wire could represent a performance and a quality metric for PCI.

## Conflicts of Interest

N.C. reports grant income from HeartFlow, Boston Scientific, Haemonetics and Beckman Coulter and speaker fees/consultancy from HeartFlow, Shockwave, Cordis and Abbott Vascular. The other authors declare no conflicts of interest.

## Supporting information


Supporting File 1

Supporting File 2

Supporting File 3

Supporting File 4

Supporting File 5

Supporting File 6

Supporting File 7

Supporting File 8

Supporting File 9

## Data Availability

The data used for the purpose of this study are only available to designated researchers and cannot be shared with other researchers.
