# Supplementary material for: Use of Intracoronary Pressure Wire Assessment and Association With Outcomes: Insights From the British Cardiovascular Intervention Society National Database
Source: Catheter Cardiovasc Interv. 2026 Aug 30;108(4):1743–53. doi: 10.1002/ccd.70835 (PMC13667319; doi:10.1002/ccd.70835)
Supplement: Supplementary file 1 [file CCD-108-1743-s001.docx]

***Supplementary Table 3. Predictors of coronary physiological assessment in patients with stable angina.***

| Predictor | | aOR [95% CI] | | | | *P*-value |  |
| --- | --- | --- | --- | --- | --- | --- | --- |
|  |  |  |  |  |  |  |  |
| Age group (years) | |  | | | |  |  |
| <60 | | Reference | | | |  |  |
| 60–69 | | 0.95 [0.92–0.99] | | | | 0.017 |  |
| 70–79 | | 0.85 [0.81–0.88] | | | | <0.001 |  |
| ≥80 | | 0.64 [0.61–0.69] | | | | <0.001 |  |
| Male | | 1.08 [1.04–1.12] | | | | <0.001 |  |
| Previous MI | | 0.97 [0.93–1.01] | | | | 0.123 |  |
| Previous CABG | | 0.84 [0.77–0.91] | | | | <0.001 |  |
| Previous PCI | | 1.23 [1.18–1.28] | | | | <0.001 |  |
| DM | | 1.00 [0.96–1.04] | | | | 0.918 |  |
| Smoking history | | 1.05 [1.02–1.08] | | | | 0.003 |  |
| Family history of CAD | |  | | | |  |  |
| None | | Reference | | | |  |  |
| Known | | 1.07 [1.03–1.10] | | | | <0.001 |  |
| Unknown | | 1.04 [0.97–1.11] | | | | 0.246 |  |
| Dyslipidaemia | | 1.06 [1.02–1.09] | | | | 0.002 |  |
| Hypertension | | 1.06 [1.02–1.10] | | | | 0.001 |  |
| PVD | | 0.94 [0.87–1.02] | | | | 0.146 |  |
| Previous stroke | | 1.07 [0.98–1.16] | | | | 0.114 |  |
| Cardiac transplant | | 0.66 [0.37–1.18] | | | | 0.157 |  |
| Valvular heart disease | | 1.10 [0.98–1.22] | | | | 0.094 |  |
| Previous non-coronary cardiac surgery | | 0.97 [0.77–1.21] | | | | 0.760 |  |
| Renal function | |  | | | |  |  |
| No renal disease | | Reference | | | |  |  |
| Functional transplant | | 0.84 [0.60–1.16] | | | | 0.284 |  |
| Renal failure | | 0.72 [0.63–0.82] | | | | <0.001 |  |
| Pre-Procedure medications | |  | | | |  |  |
| Aspirin | | 1.09 [1.02–1.16] | | | | 0.007 |  |
| Clopidogrel | | 1.02 [0.97–1.07] | | | | 0.483 |  |
| Ticagrelor | | 1.03 [0.97–1.08] | | | | 0.343 |  |
| Prasugrel | | 0.83 [0.74–0.94] | | | | 0.004 |  |
| Warfarin | | 0.97 [0.85–1.10] | | | | 0.636 |  |
| Procedure characteristics | |  | | | |  |  |
| Intracoronary imaging | | 1.22 [1.16–1.27] | | | | <0.001 |  |
| Calcium modification | | 0.75 [0.71–0.80] | | | | <0.001 |  |
| GPIIb/IIIa inhibitors | | 0.90 [0.85–0.95] | | | | <0.001 |  |
| Arterial access | |  | | | |  |  |
| Femoral | | 0.75 [0.69–0.81] | | | | <0.001 |  |
| Radial | | 1.23 [1.13–1.34] | | | | <0.001 |  |
| Other | | 1.15 [0.89–1.49] | | | | 0.291 |  |
| Angiographic severity | |  | | | |  |  |
| Left main stem | |  | | | |  |  |
| Normal | | Reference | | | |  |  |
| Mild disease | | 1.00 [0.96–1.05] | | | | 0.970 |  |
| Severe disease | | 0.75 [0.67–0.85] | | | | <0.001 |  |
| Totally occluded | | 0.93 [0.50–1.70] | | | | 0.802 |  |
| Proximal LAD | |  | | | |  |  |
| Normal | | Reference | | | |  |  |
| Mild disease | | 1.30 [1.25–1.36] | | | | <0.001 |  |
| Moderate disease | | 2.43 [2.29–2.58] | | | | <0.001 |  |
| Severe disease | | 0.79 [0.75–0.85] | | | | <0.001 |  |
| Totally occluded | | 0.40 [0.35–0.47] | | | | <0.001 |  |
| Other LAD | |  | | | |  |  |
| Normal | | Reference | | | |  |  |
| Mild disease | | 1.06 [1.01–1.11] | | | | 0.029 |  |
| Moderate disease | | 2.10 [1.99–2.22] | | | | <0.001 |  |
| Severe disease | | 0.82 [0.77–0.87] | | | | <0.001 |  |
| Totally occluded | | 0.36 [0.32–0.40] | | | | <0.001 |  |
| LCx | |  | | | |  |  |
| Normal | | Reference | | | |  |  |
| Mild disease | | 1.01 [0.96–1.05] | | | | 0.764 |  |
| Moderate disease | | 1.49 [1.41–1.58] | | | | <0.001 |  |
| Severe disease | | 0.76 [0.71–0.81] | | | | <0.001 |  |
| Totally occluded | | 0.73 [0.66–0.80] | | | | <0.001 |  |
| RCA | |  | | | |  |  |
| Normal | | Reference | | | |  |  |
| Mild disease | | 1.11 [1.06–1.16] | | | | <0.001 |  |
| Moderate disease | | 1.99 [1.88–2.11] | | | | <0.001 |  |
| Severe disease | | 0.85 [0.80–0.91] | | | | <0.001 |  |
| Totally occluded | | 0.49 [0.45–0.53] | | | | <0.001 |  |
|  | Attempted vessels | |  |  |  | | |
|  | Left main stem | | 0.80 [0.71–0.89] | | <0.001 | | |
|  | Proximal LAD | | 1.43 [1.35–1.51] | | <0.001 | | |
|  | Other LAD | | 1.88 [1.79–1.98] | | <0.001 | | |
|  | LCx | | 1.42 [1.34–1.51] | | <0.001 | | |
|  | RCA | | 1.21 [1.14–1.28] | | <0.001 | | |
|  | Graft | | 0.91 [0.75–1.11] | | 0.368 | | |

**Abbreviations**: aOR – Adjusted Odds ratio, CABG – Coronary Artery Bypass Graft, CAD – Coronary Artery Disease, DM – Diabetes Mellitus, LAD – Left Anterior Descending Artery, LCx – Left Circumflex Artery, LV – Left Ventricle, MI – Myocardial Infarction, PCI – Percutaneous Coronary Intervention, PVD – Peripheral Vascular Disease, RCA – Right Coronary Artery.
