# Supplementary material for: Use of Intracoronary Pressure Wire Assessment and Association With Outcomes: Insights From the British Cardiovascular Intervention Society National Database
Source: Catheter Cardiovasc Interv. 2026 Aug 30;108(4):1743–53. doi: 10.1002/ccd.70835 (PMC13667319; doi:10.1002/ccd.70835)
Supplement: Supplementary file 2 [file CCD-108-1743-s003.docx]

***Supplementary Table 2. Baseline and procedural characteristics in patients with stable angina.***

| Characteristics, % | | No physiological assessment (N=115,459; 84.49%) | Physiological assessment (N=21,190; 15.51%) | | *P*-value | |  |
| --- | --- | --- | --- | --- | --- | --- | --- |
|  |  |  |  |  |  |  |  |
| Age group (years) | |  |  | | <0.001 | |  |
| <60 | | 28.23 | 32.18 | |  | |  |
| 60–69 | | 31.91 | 33.99 | |  | |  |
| 70–79 | | 28.75 | 26.31 | |  | |  |
| ≥80 | | 11.12 | 7.52 | |  | |  |
| Male | | 76.03 | 77.64 | | <0.001 | |  |
| Race/Ethnicity | |  |  | |  | |  |
| Caucasian | | 59.94 | 56.69 | |  | |  |
| Black | | 0.57 | 0.40 | |  | |  |
| Asian | | 5.99 | 5.03 | |  | |  |
| Oriental | | 0.15 | 0.16 | |  | |  |
| Other | | 6.75 | 6.47 | |  | |  |
| Unknown | | 26.60 | 31.26 | |  | |  |
| Previous MI | | 35.02 | 33.97 | | 0.003 | |  |
| Previous CABG | | 11.64 | 4.30 | | <0.001 | |  |
| Previous PCI | | 39.05 | 43.01 | | <0.001 | |  |
| DM | | 23.65 | 22.80 | | 0.007 | |  |
| Smoking history | | 55.80 | 57.25 | | <0.001 | |  |
| Family history of CAD | |  |  | | <0.001 | |  |
| None | | 48.77 | 46.25 | |  | |  |
| Known | | 45.33 | 47.89 | |  | |  |
| Unknown | | 5.89 | 5.86 | |  | |  |
| Dyslipidaemia | | 63.00 | 64.64 | | <0.001 | |  |
| Hypertension | | 63.25 | 63.47 | | 0.547 | |  |
| PVD | | 4.50 | 3.73 | | <0.001 | |  |
| Previous stroke | | 3.79 | 3.83 | | 0.813 | |  |
| Cardiac transplant | | 0.12 | 0.06 | | 0.017 | |  |
| Valvular heart disease | | 2.47 | 2.17 | | 0.008 | |  |
| Previous non-coronary cardiac surgery | | 0.54 | 0.47 | | 0.235 | |  |
| Renal function | |  |  | | <0.001 | |  |
| No renal disease | | 97.43 | 98.36 | |  | |  |
| Functional transplant | | 0.27 | 0.21 | |  | |  |
| Renal failure | | 2.30 | 1.43 | |  | |  |
| LV ejection fraction | |  |  | |  | |  |
| Good (>50%) | | 52.54 | 56.03 | |  | |  |
| Moderate (30%–50%) | | 10.00 | 8.39 | |  | |  |
| Poor (<30%) | | 2.44 | 1.73 | |  | |  |
| Unknown | | 35.02 | 33.86 | |  | |  |
| Pre-Procedure medications | |  |  | |  | |  |
| Aspirin | | 87.43 | 88.67 | | <0.001 | |  |
| Clopidogrel | | 73.52 | 73.27 | | 0.453 | |  |
| Ticagrelor | | 13.70 | 15.24 | | <0.001 | |  |
| Prasugrel | | 1.84 | 1.80 | | 0.666 | |  |
| Warfarin | | 1.63 | 1.48 | | 0.119 | |  |
| Procedure characteristics | |  |  | |  | |  |
| Intracoronary imaging | | 12.53 | 15.17 | | <0.001 | |  |
| Calcium modification | | 9.71 | 7.41 | | <0.001 | |  |
| GPIIb/IIIa inhibitors | | 10.76 | 9.85 | | <0.001 | |  |
| Stent thrombosis | |  |  | |  | |  |
| Present | | 0.31 | 0.27 | |  | |  |
| Unknown | | 36.86 | 31.49 | |  | |  |
| Arterial access | |  |  | |  | |  |
| Femoral | | 26.86 | 15.88 | | <0.001 | |  |
| Radial | | 75.64 | 85.19 | | <0.001 | |  |
| Other | | 0.44 | 0.37 | | 0.165 | |  |
| Angiographic severity | |  |  | |  | |  |
| Left main stem | |  |  | | <0.001 | |  |
| Normal | | 78.33 | 78.83 | |  | |  |
| Mild disease | | 15.45 | 17.97 | |  | |  |
| Severe disease | | 5.92 | 3.15 | |  | |  |
| Totally occluded | | 0.30 | 0.06 | |  | |  |
| Proximal LAD | |  |  | | <0.001 | |  |
| Normal | | 34.56 | 27.78 | |  | |  |
| Mild disease | | 23.96 | 27.52 | |  | |  |
| Moderate disease | | 7.52 | 20.47 | |  | |  |
| Severe disease | | 28.21 | 23.12 | |  | |  |
| Totally occluded | | 5.75 | 1.11 | |  | |  |
| Other LAD | |  | |  | | <0.001 | |
| Normal | | 33.82 | | 23.90 | |  | |
| Mild disease | | 22.45 | | 19.94 | |  | |
| Moderate disease | | 8.92 | | 22.77 | |  | |
| Severe disease | | 28.73 | | 31.77 | |  | |
| Totally occluded | | 6.08 | | 1.61 | |  | |
| LCx | |  | |  | | <0.001 | |
| Normal | | 36.51 | | 33.74 | |  | |
| Mild disease | | 23.11 | | 26.50 | |  | |
| Moderate disease | | 7.55 | | 13.31 | |  | |
| Severe disease | | 27.31 | | 23.27 | |  | |
| Totally occluded | | 5.52 | | 3.19 | |  | |
| RCA | |  | |  | | <0.001 | |
| Normal | | 30.28 | | 30.16 | |  | |
| Mild disease | | 19.98 | | 25.88 | |  | |
| Moderate disease | | 6.30 | | 13.78 | |  | |
| Severe disease | | 30.82 | | 25.31 | |  | |
| Totally occluded | | 12.62 | | 4.87 | |  | |
|  | Attempted vessels |  |  | |  | |  |
|  | Left main stem | 6.57 | 3.91 | | <0.001 | |  |
|  | Proximal LAD | 31.94 | 38.70 | | <0.001 | |  |
|  | Other LAD | 30.22 | 44.97 | | <0.001 | |  |
|  | LCx | 25.78 | 26.44 | | 0.045 | |  |
|  | RCA | 34.53 | 29.34 | | <0.001 | |  |
|  | Graft | 2.46 | 0.63 | | <0.001 | |  |

**Abbreviation**: CABG – Coronary Artery Bypass Graft, CAD – Coronary Artery Disease, DM – Diabetes Mellitus, LAD – Left Anterior Descending Artery, LCx – Left Circumflex Artery, LV – Left Ventricle, MI – Myocardial Infarction, PCI – Percutaneous Coronary Intervention, PVD – Peripheral Vascular Disease, RCA – Right Coronary Artery.
