# Supplementary material for: Use of Intracoronary Pressure Wire Assessment and Association With Outcomes: Insights From the British Cardiovascular Intervention Society National Database
Source: Catheter Cardiovasc Interv. 2026 Aug 30;108(4):1743–53. doi: 10.1002/ccd.70835 (PMC13667319; doi:10.1002/ccd.70835)
Supplement: Supplementary file 3 [file CCD-108-1743-s004.docx]

***Supplementary Table 5. Adjusted outcomes in patients with stable angina.***

| Hospital outcomes | | | Physiological assessment aOR [95% CI] | *P*-value | |
| --- | --- | --- | --- | --- | --- |
|  | MACCE | 0.90 [0.71–1.14] | | | 0.371 |
|  | Mortality | 0.59 [0.35–1.01] | | | 0.056 |
|  | Stroke/TIA | 1.52 [0.82–2.82] | | | 0.181 |
|  | New infarction | 0.92 [0.69–1.23] | | | 0.589 |
|  | Major bleed | 0.92 [0.61–1.39] | | | 0.692 |
|  | Re-intervention | 1.05 [0.69–1.60] | | | 0.809 |
|  | Procedural complications | 0.90 [0.83–0.98] | | | 0.013 |
|  | Arterial access complications | 0.54 [0.43–0.69] | | | <0.001 |
|  | Two or more stents | 0.74 [0.71–0.77] | | | <0.001 |
|  | Three or more stents | 0.66 [0.63–0.69] | | | <0.001 |
|  | Four or more stents | 0.61 [0.56–0.65] | | | <0.001 |

*Reference group: No physiological assessment.

**Abbreviations**: aOR – Adjusted Odds ratio, CI – Confidence Interval, MACCE – Major Adverse Cardiovascular and Cerebrovascular Events, TIA – Transient Ischemic Attack.
