# Supplementary material for: Use of Intracoronary Pressure Wire Assessment and Association With Outcomes: Insights From the British Cardiovascular Intervention Society National Database
Source: Catheter Cardiovasc Interv. 2026 Aug 30;108(4):1743–53. doi: 10.1002/ccd.70835 (PMC13667319; doi:10.1002/ccd.70835)
Supplement: Supplementary file 4 [file CCD-108-1743-s005.docx]

***Supplementary Table 4. Unadjusted in-hospital outcomes in patients with stable angina.***

| Outcomes, % | No physiological assessment (N=115,459; 84.49%) | Physiological assessment (N=21,190; 15.51%) | *P*-value |
| --- | --- | --- | --- |
| MACCE | 0.51 | 0.41 | 0.054 |
| Mortality | 0.18 | 0.07 | <0.001 |
| Stroke/TIA | 0.05 | 0.07 | 0.356 |
| New infarction | 0.30 | 0.28 | 0.763 |
| Major bleed | 0.19 | 0.13 | 0.066 |
| Re-intervention | 0.13 | 0.14 | 0.772 |
| Procedural complications | 3.84 | 3.46 | 0.009 |
| Arterial access complications | 0.82 | 0.36 | <0.001 |
| Two or more stents | 47.98 | 44.88 | <0.001 |
| Three or more stents | 19.26 | 15.72 | <0.001 |
| Four or more stents | 6.77 | 4.98 | <0.001 |

**Abbreviations:** MACCE – Major Adverse Cardiovascular and Cerebrovascular Events, TIA – Transient Ischemic Attack.
