# Supplementary material for: Use of Intracoronary Pressure Wire Assessment and Association With Outcomes: Insights From the British Cardiovascular Intervention Society National Database
Source: Catheter Cardiovasc Interv. 2026 Aug 30;108(4):1743–53. doi: 10.1002/ccd.70835 (PMC13667319; doi:10.1002/ccd.70835)
Supplement: Supplementary file 5 [file CCD-108-1743-s007.docx]

***Supplementary Figure 1. Study flow diagram.***

***Supplementary Figure 2. Utilization trends of coronary physiology and intracoronary imaging during PCI among patients with stable angina.***

***Supplementary Figure 3. Utilization trends of coronary physiology stratified by the presence of guideline recommendation for coronary physiological assessment (I.e., the presence of moderate disease) among stable angina patients.***
