# Supplementary material for: Use of Intracoronary Pressure Wire Assessment and Association With Outcomes: Insights From the British Cardiovascular Intervention Society National Database
Source: Catheter Cardiovasc Interv. 2026 Aug 30;108(4):1743–53. doi: 10.1002/ccd.70835 (PMC13667319; doi:10.1002/ccd.70835)
Supplement: Supplementary file 6 [file CCD-108-1743-s009.docx]

**Supplementary Table 1. Angiographic disease severity.**

| Angiographic severity |  |  | |  | |  |
| --- | --- | --- | --- | --- | --- | --- |
| Left main stem |  |  | | <0.001 | |  |
| Normal | 77.16 | 77.61 | |  | |  |
| Mild disease | 16.42 | 19.01 | |  | |  |
| Severe disease | 6.09 | 3.33 | |  | |  |
| Totally occluded | 0.33 | 0.06 | |  | |  |
| Proximal LAD |  |  | | <0.001 | |  |
| Normal | 32.92 | 27.08 | |  | |  |
| Mild disease | 25.53 | 28.51 | |  | |  |
| Moderate disease | 7.67 | 20.46 | |  | |  |
| Severe disease | 28.35 | 22.78 | |  | |  |
| Totally occluded | 5.54 | 1.17 | |  | |  |
| Other LAD |  | |  | | <0.001 | |
| Normal | 31.21 | | 22.61 | |  | |
| Mild disease | 23.68 | | 20.54 | |  | |
| Moderate disease | 9.90 | | 23.32 | |  | |
| Severe disease | 29.09 | | 31.67 | |  | |
| Totally occluded | 6.12 | | 1.86 | |  | |
| LCx |  | |  | | <0.001 | |
| Normal | 33.16 | | 30.91 | |  | |
| Mild disease | 23.13 | | 25.77 | |  | |
| Moderate disease | 7.93 | | 13.62 | |  | |
| Severe disease | 29.25 | | 25.99 | |  | |
| Totally occluded | 6.53 | | 3.71 | |  | |
| RCA |  | |  | | <0.001 | |
| Normal | 27.42 | | 27.80 | |  | |
| Mild disease | 21.09 | | 25.95 | |  | |
| Moderate disease | 7.00 | | 14.19 | |  | |
| Severe disease | 31.93 | | 26.70 | |  | |
| Totally occluded | 12.55 | | 5.35 | |  | |

**Abbreviations:** LAD – Left Anterior Descending Artery, LCx – Left Circumflex Artery, RCA – Right Coronary Artery.
